# Serum adipsin levels in rheumatic diseases: defining its role in disease activity and progression in rheumatoid arthritis and axial spondyloarthritis

**DOI:** 10.3389/fimmu.2025.1636158

**Published:** 2025-08-06

**Authors:** Marta Novella-Navarro, Laura González-Sánchez, Borja Hernández-Breijo, Rebeca Pérez De Diego, Pilar Sánchez-Corral, Margarita López-Trascasa, Maria-Eugenia Miranda-Carús, Alejandro Villalba, Alejandro Balsa, Eugenio De Miguel, Chamaida Plasencia-Rodríguez, Fernando Corvillo

**Affiliations:** ^1^ Rheumatology Department, Hospital Universitario La Paz, Madrid, Spain; ^2^ Immuno-Rheumatology Investigation Group, IdiPAZ, Hospital Universitario La Paz, Madrid, Spain; ^3^ Complement Research Group, Hospital La Paz Institute for Health Research (IdiPAZ), La Paz University Hospital, Madrid, Spain; ^4^ Center for Biomedical Network Research on Rare Diseases, Madrid, Spain; ^5^ Laboratory of Immunogenetics of Human Diseases, IdiPAZ, Hospital Universitario La Paz, Madrid, Spain; ^6^ Departamento de Medicina, Universidad Autónoma de Madrid, Madrid, Spain

**Keywords:** adipsin, rheumatoid arthritis, rheumatic disease, obesity, adipokines

## Abstract

Rheumatoid arthritis (RA) and axial spondyloarthritis (axSpA) are the most common rheumatic diseases (RDs). They are characterised by chronic inflammation of the joints and musculoskeletal components. Adipose tissue releases adipokines that regulate numerous biological processes, including inflammation, thus stabilising the relationship between RDs and adipokines, such as adiponectin and leptin. The correlation between these adipokines and RA highlights a possible role of other adipokines in RDs. Therefore, we decided to analyse the role of the adipokine adipsin in the context of RDs. Adipsin levels were measured in serum from 233 patients (66 early-RA, 98 established-RA, and 69 axSpA) and 88 healthy controls (HCs). Associations between adipsin and clinical or demographic variables were assessed using univariate and multivariate regression models. The diagnostic utility of adipsin was evaluated using ROC curve analysis. Our study revealed that adipsin concentrations were significantly higher in both early-RA and established-RA patients than in axSpa and in HCs. No significant differences were found between axSpA and HCs. In early-RA, female sex and prednisone use were independently associated with higher adipsin levels. In established-RA, age and disease duration showed a positive association with adipsin concentrations. In axSpA, disease duration and CRP correlated with adipsin levels, but no consistent associations were observed for BMI or HLA-B27 status. ROC analysis revealed good discriminatory capacity of adipsin to differentiate early-RA from HCs (AUC = 0.82; optimal cut-off: 1.325 µg/mL). We provide evidence supporting the involvement of adipsin in the pathophysiology of RA and highlight its value as a new potential biomarker.

## Introduction

Rheumatoid arthritis (RA) and axial spondyloarthritis (axSpA) are the most common rheumatic diseases (RDs). Both are characterised by chronic inflammation of the joints and musculoskeletal components and significantly diminish the quality of life and life expectancy of affected individuals. The risk of developing RDs is associated with genetic and epigenetic factors, immune system abnormalities (in the form of autoantibody production), and environmental and lifestyle conditions (1, 2). Smoking and obesity are modifiable lifestyle conditions that play a key role in RD. Notably, high body mass index (BMI) is associated with more severe symptoms and more frequent disability in patients with RDs and can lead to higher disease activity and poorer responses to anti-TNF treatments, suggesting that obesity may influence their efficacy (3–5).

Progressive and excessive fat accumulation in obesity leads to substantial changes in the quantity and phenotype of immune cells residing in white adipose tissue, and contributes to maintenance of a pro-inflammatory status. Adipose tissue acts as an endocrine organ, releasing adipokines that regulate numerous biological processes, including inflammation. In obesity, the adipokine profile shifts towards increased pro-inflammatory factors, such as leptin, and decreased anti-inflammatory factors, such as adiponectin (6). Previous studies have shown that serum levels of adiponectin and leptin are elevated in RA, and that leptin is associated with disease activity (4, 7, 8).

Adipsin, also known as complement factor D (FD), is an adipokine predominantly secreted by adipocytes, playing essential roles in both immune function and metabolic homeostasis. It is a serine protease that serves as a rate-limiting enzyme in the activation of the alternative pathway of the complement system (9).

The complement system is a fundamental component of innate immunity, contributing to host defence against infections, modulating adaptive immune responses, and facilitating the clearance of cellular debris. It can be activated through three distinct pathways: the classical, lectin, and alternative pathways. Despite their differences, all three converge on the formation of the C3 convertase enzyme complex (C3bBb), which cleaves complement component C3 into C3a and C3b. C3b promotes pathogen clearance via opsonization and contributes to the formation of C5 convertase, ultimately leading to terminal pathway activation and cell lysis. Meanwhile, C3a and C5a act as potent anaphylatoxins that enhance inflammation. Adipsin is critical for the activation of the alternative pathway through its cleavage of complement factor B, but only when factor B is bound to C3b. This interaction leads to the formation of C3bBb, which amplifies the immune response by catalysing further generation of C3a and C3b. Beyond its immunological functions, the adipsin/C3a axis is implicated in a range of physiological processes, including immune regulation, cell migration, insulin sensitivity, adipocyte differentiation, and the homing of hematopoietic stem cells (9).Serum adipsin levels in healthy individuals are in the range of 1-2 µg/mL (10), and while they are lower in conditions such as insulin resistance (11, 12), adipsin levels increase in obesity and other metabolic disorders, demonstrating the importance of this adipokine in both health and disease (13–16). Preliminary research suggests a connection between adipsin levels and disease activity in early-RA (8). However, previous studies have reported no significant differences in adipsin levels between early and established stages of the disease (17), leaving the precise functional consequences of adipsin elevation in the context of RA speculative. Additionally, Valverde-Franco et al. found that serum adipsin levels were increased in humans and mouse models of osteoarthritis and significantly associated with greater cartilage volume loss (18). Other research has demonstrated a prominent role for adipsin in the pathogenesis of arthritis in mice (19). Therefore, the main objectives of our study were to analyse serum adipsin levels in RA and axSpA patients with active disease, and to identify potential differences between the two groups. As a secondary objective, we compared adipsin levels in RA patients at different stages of the disease (early vs established disease).

## Materials and methods

### Ethics approval and consent to participate

The study was approved by the Ethics Committee of La Paz University Hospital (study code: PI-4596) and conducted according to the guidelines of the 1975 Declaration of Helsinki. Spanish legal requirements for the protection of confidential data were adhered to. All the patients and healthy controls signed a written informed consent document before inclusion in the study.

### Patients and biological samples

The study population comprised 233 patients (66 early-RA, 98 established-RA, and 69 axSpA) aged ≥18 years from the RA-PAZ (20) and SpA-PAZ (21) cohorts. Clinical data were systematically collected in a database through an electronic case report form at the Biologic Unit of La Paz University Hospital. Early-RA and established-RA patients fulfilled the 2010 criteria for RA of the American College of Rheumatology/European League Against Rheumatism (22). Patients with axSpA fulfilled the criteria of the Assessment of Spondyloarthritis international Society (23). Disease duration was <6 months in early-RA patients, who, in addition, did not have concomitant autoimmune/inflammatory or infectious diseases capable of interfering with the interpretation of the results. Serum samples from early-RA patients were obtained at the first visit to the rheumatology department before starting conventional synthetic disease-modifying antirheumatic drugs (csDMARDs) therapy. Established-RA and axSpA patients were recruited before starting biological therapy, and had moderate or high disease activity (28-joint Disease Activity Score [DAS28] >3.2, Axial Spondyloarthritis Disease Activity Score [ASDAS] >2.1) at the time of inclusion. We also included 88 healthy controls (HCs). Serum samples from patients and HCs were frozen immediately and stored in La Paz University Hospital Biobank.

### Measurement of adipsin levels

Serum levels of adipsin (complement FD) were measured using an in-house enzyme-linked immunosorbent assay (ELISA). ELISA plates (MaxiSorp^®^, Nunc) were coated with 100 ng/well of a mouse monoclonal anti-human FD antibody (GAU 01-04-02, Invitrogen, Carlsbad, CA, USA) diluted in carbonate-bicarbonate buffer pH 9.3 (overnight, 4°C). Plates were washed four times in phosphate-buffered saline (PBS) 0.1% Tween 20 and then blocked with 150 µL/well of PBS 1% bovine serum albumin (BSA) for 1 hour at 37°C. A standard curve ranging from 0 to 80 ng/mL of purified FD (Complement Technology, Tyler, TX, USA) was run on each plate, alongside with an internal control serum sample used to assess that the inter-assay variability coefficient of variation was below 10%. After washing, serum samples were diluted in PBS-BSA 0.1% (containing 1 µg/mL of human intravenous IgG) at 1/50, 1/100, and 1/200 dilutions for analysis. IgG was included in the PBS-1% BSA buffer to prevent interference by rheumatoid factors present in serum samples from early-RA and RA patients. After incubation for 1 hour at 37°C, the plates were washed as described above. FD was detected using a mouse monoclonal anti-human FD antibody conjugated with biotin (GAU 008-01B-02, Invitrogen) (1/2500 dilution, 1 hour at 37°C). After washing, the plates were incubated with streptavidin-peroxidase polymer (S2438, Sigma Aldrich, St Louis, MO, USA) (1/400, 45 minutes at 37°C). The coloured reactions were developed using ABTS as substrate, and read at 405/620 nm in an Epoch™ Microplate Spectrophotometer (BioTek Instruments, Inc., Winooski, VT, USA). All samples were measured in duplicate.

### Statistical analyses

Descriptive analyses were performed for the demographic and clinical variables. The results are shown as mean ± standard deviation or median and interquartile range, depending on the data distribution, for continuous variables and relative frequencies for categorical variables. The frequency data were compared using Pearson’s chi-square test or Fisher’s exact test. Unpaired continuous data were compared using the unpaired *t* test or Mann-Whitney test, depending on the data distribution. The Kruskal-Wallis test was used to perform and Dunn’s multiple comparisons and *post-hoc* corrections with Dunn-Bonferroni test were performed to analyse differences in adipsin concentrations between the groups.

To evaluate the association of the different clinical variables with adipsin levels in the four groups of patients, bivariate and multivariate linear regression models were performed.

To evaluate the discriminative capacity of adipsin levels, a Receiver Operating Characteristic (ROC) and Precision-Recall (PR) curves was constructed. This analysis makes it possible to determine the sensitivity and specificity of the marker at different cut-off points calculated by Youden Index, as well as to calculate the area under the curve (AUC), which reflects its overall performance in differentiating between healthy controls and different groups of patient.

The analysis was performed using IBM SPSS Statistics, Version 24.0 (IBM Corp., Armonk, NY, USA) and R statistics 4.4.2. A *P* value <0.05 was considered statistically significant. The graphs were constructed using GraphPad Prism version 9 (GraphPad Software, San Diego, CA, USA).

## Results

### Patient and disease characteristics

A total of 233 patients (66 with early-RA, 98 with established-RA, and 69 with axSpA), and 88 HCs were included in this study. Demographics and disease characteristics are summarised in **Table 1**. Patients with axSpA were slightly younger than HCs (*P*<0.05), early-RA (*P*<0.05), and RA patients (*P*<0.01), while there were no age differences between HCs, early-RA patients, and established-RA patients. Moreover, only 41% of axSpA patients were female, in contrast to HCs (76%), early-RA patients (70%), and established-RA patients (81%). Regarding the use of concomitant therapies, established-RA patients more frequently received prednisone or csDMARDs. Early-RA patients had low rheumatoid factor and anti–citrullinated protein antibody titers and a lower DAS28 score at sample collection than RA patients (*P*<0.01, in all cases). C-reactive protein was lower in early-RA patients than in established-RA or axSpA patients (*P*<0.01, in both comparisons). There were no differences between the groups in terms of BMI.

**Table 1:** Characteristics of the study population.

Characteristics	Healthy controls (n=88)	Early-RA (n=66)	Established-RA (n=98)	axSpA (n=69)
Age (years)	53 ± 11 ($)	57 ± 13 ($)	57 ± 13 ($$)	48 ± 13
Female	67 (76) ($$$)	46 (70) ($$$)	79 (81) ($$$)	28 (41)
Smokers	–	32 (48)	45 (46)	32 (52)
Disease duration (years)	–	–	9 (4-15)	7 (3-16)
RF	–	36 (54) (##)	75 (77)	–
ACPA	–	40 (60) (##)	78 (80)	–
HLA-B27	–	–	–	49 (72)
BMI (kg/m^2^)	–	27.0 ± 4.4	26.6 ± 4.9	27.1 ± 5.0
BMI≥25 kg/m^2^	–	42 (68)	58 (59)	41 (59)
DAS28	–	4.4 ± 1.4 (##)	4.9 ± 1.3	–
ASDAS	–	–	–	3.5 ± 1.0
CRP (mg/L)	–	1 (0-3) (###,$$$)	6 (2-20)	8 (3-24)
csDMARDs	–	4 (6) (###,$$$)	91 (93) ($$$)	40 (58)
Prednisone	–	20 (31) (##,$)	57 (58) ($$$)	9 (13)

The table shows the mean ± SD, median (IQR) or absolute number (percentage) of the four groups included in the study (n=321). A *P* value<0.05 was considered statistically significant. Difference with early-RA. #Difference with established-RA. $Difference with axSpA ($: *P*<0.05; ##, $$: *P*<0.01; ###, $$$: *P*<0.0001). ACPA, anti–citrullinated peptide antibody; ASDAS, Ankylosing Spondylitis Disease Activity Index; BMI, body mass index; CRP, C-reactive protein; csDMARD, conventional synthetic disease-modifying antirheumatic drug; DAS28, 28-joint disease activity score; HLA-B27, human leukocyte antigen B27; RA, rheumatoid arthritis; RF, rheumatoid factor.

### Differences in adipsin concentrations by rheumatic disease

**Figure 1:**
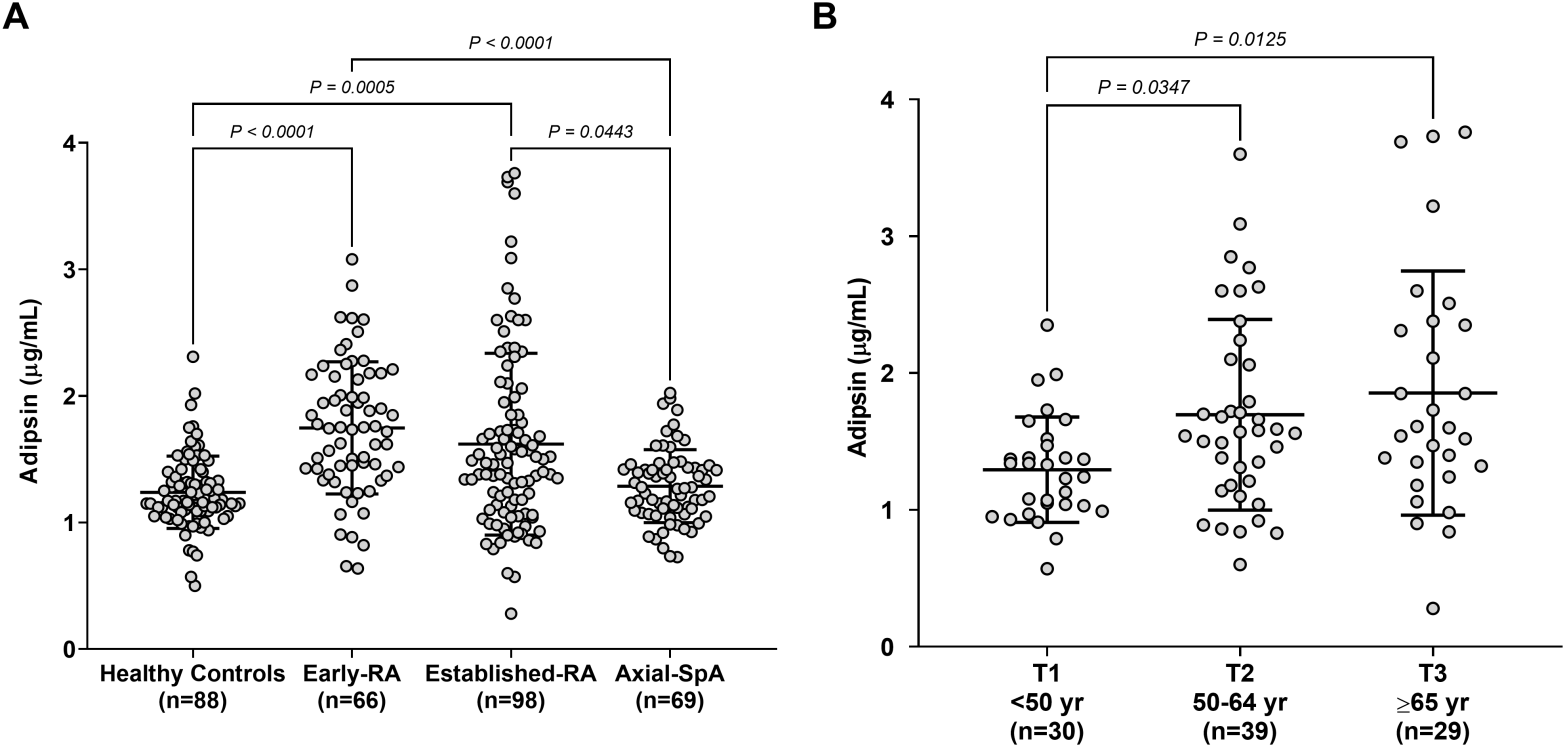
Serum adipsin levels. **(A)** Adipsin concentrations were measured in the serum samples of the 4 cohorts included (HCs, early-RA, established-RA, and axSpA). Differences in adipsin concentrations between the groups were analysed using the Kruskal-Wallis test plus Dunn’s multiple comparisons test. A *P* value <0.05 was considered statistically significant. RA, rheumatoid arthritis; SpA, spondyloarthritis. No statistically significant differences were observed between HCs and axSpa, and between early-RA and established-RA patients. **(B)** Adipsin concentrations were analysed and compared by age tertiles (T1, <50 years; T2, 50–64 years; T3, ≥65 years) in established-RA patients (n=98). Differences in adipsin concentrations between the groups were analysed using the Kruskal-Wallis test plus Dunn’s multiple comparisons test. A *P* value <0.05 was considered statistically significant.

Median (IQR) serum adipsin concentrations (µg/mL) were as follows: HCs, 1.2 (1.1-1.3); early-RA, 1.7 (1.4-2.2); established-RA, 1.5 (1.1-1.9); axSpA, 1.3 (1.1-1.4). Adipsin concentrations in axSpA patients were similar to those in HCs (*P* = 1.0). Both early-RA and established-RA patients had significantly higher adipsin concentrations compared to axSpA (*P* < 0.0001 and *P* = 0.0443, respectively). Similarly, early-RA and established-RA patients showed significantly higher levels than HCs (*P* < 0.0001 and *P* = 0.0005, respectively). Although adipsin concentrations were slightly higher in early-RA than in established-RA patients, this difference did not reach statistical significance. These results are summarised in **Figure 1A**.

### Association between demographic and disease characteristics and adipsin concentrations in different groups of patients

In order to gain further insight into differences in adipsin concentration between the groups, we investigated the association with demographic and clinical parameters (**Table 2**). Among the variables collected for HCs, adipsin concentration was higher in males 1.3 (1.2-1.5) µg/mL than in females 1.1 (1.0-1.3) µg/mL, with a significant association (B = 0.16; 95%CI [0.02 – 0.30).

**Table 2:** Association between clinical features and adipsin levels for each group of patients. Linear regression models for bivariate and multivariate analysis.

	Healthy controls (n=88)		Early-RA (n=66)		Established-RA (n=98)		axSpa (n=69)	
Variable	Univariate	Multivariate	Univariate	Multivariate	Univariate	Multivariate	Univariate	Multivariate
Age (years)	-0.002 (-0.007-0.004)	_	0.01 (-0.003-0.02)	–	**0.02** (0.01-0.03)*	**0.01** (0.007-0.028)*	0.004 (-0.001-0.10)	–
Sex (ref male)	**0.16** (0.02-0.30)*	–	**0.48** (0.04-0.91)*	**0.46** (0-42-0.88)*	0.16 (-0.20-0.56)	–	0.05 (-0.08-0.19)	–
BMI (kg/m2)	–	–	0.008(-0.04-0.05)	–	0.02 (-0.00-0.05)	–	0.007 (-0.007-0.021)	–
Disease duration (years)	–	–	–	–	**0.02** (0.01-0.04)*	**0.02** (0.005-0.036)*	**0.011** (0-005-0.18)*	–
RF	–	–	0.06 (-0.48-0.34)	–	-0.21 (-0.56-0.12)	–	–	–
ACPA	–	–	-0.25 (-0.67-0.16)	–	0.13 (-0.23-0.50)	–	–	–
HLA-B27	–	–	–	–	–	–	0.04 (-0.10-0.19)	–
CRP	–	–	-0.006 (-0.21-0.008)	–	0.00 (-0.009-0.009)	–	0.003* **(0.000-0.005)**	–
DAS28	–	–	0.02 (-0.13-0.18)	–	-0.07 (-0.19-0.03)	–	–	–
ASDAS	–	–	–	–	–	–	0.06 (-0.007-0.04)	–
csDMARDs	–	–	–	–	-0.32 (-0.88-0.23)	–	0.09 (-0.04-0.23)	–
Prednisone (ref yes)	–	–	**0.55** (0.12-0.99)*	**0.52** (0.10-0.95)*	-0.02 (-0.31-0.26)	–	–	–

Data of univariate and multivariate analysis of linear regression are expressed by B coefficient and 95% Confidence intervals. Results marked in bold and * are statistically significant. ACPA, anti–citrullinated peptide antibody; ASDAS, Ankylosing Spondylitis Disease Activity Index; BMI, body mass index; CRP, C-reactive protein; csDMARD, conventional synthetic disease-modifying antirheumatic drug; DAS28, 28-joint Disease Activity Score; HLA-B27, human leukocyte antigen B27; RA, rheumatoid arthritis; RF, rheumatoid factor; axSpA, axial spondyloarthritis.

To explore the potential influence of clinical and demographic variables on adipsin levels, we performed both univariate and multivariate linear regression analyses in each patient group. In early RA patients, sex and prednisone use were significantly associated with adipsin levels. Female sex was linked to higher adipsin levels (in both univariate and multivariate models (B = 0.48 95%CI [0.04–0.91], and B = 0.46 95%CI[0.42–0.88], respectively). Additionally, prednisone use was associated with increased adipsin levels in early RA compared to those without the treatment (2.0 [1.5-2.5] µg/mL vs 1.6 [1.4-2.0] µg/mL) (B= 0.55 95%CI [0.12–0.99], and B = 0.52 95%CI[0.10–0.95]).

In established RA, age and disease duration showed a significant association with adipsin levels in both models. Age was positively associated (B = 0.02 [0.01–0.03]; multivariate B = 0.01 [0.007–0.028]), as was disease duration (B = 0.02 [0.01–0.04]; multivariate B = 0.02 [0.005–0.036]). Adipsin concentrations in established-RA patients were also analysed by age tertiles (T1, <50 years, n=30; T2, 50–64 years, n=39; T3, ≥65 years, n=29). Median (IQR) values were as follows: T1, 1.3 (1.0-1.5) µg/mL; T2, 1.6 (1.2-2.1) µg/mL; T3, 1.6 (1.3-2.4) µg/mL. We found that adipsin concentration was significantly lower in T1 (T1 vs. T2, P=0.0347; T1 vs. T3, P=0.0125). However, no differences were detected between T2 and T3 (**Figure 1B**).

In the Ax-SpA group, disease duration (B = 0.011 [0.005–0.018]) and CRP (B = 0.003 [0.000–0.005]) were positively associated with adipsin levels in univariate analysis. No consistent associations were observed for BMI, RF, ACPA, or HLA-B27 status.

### Predictive values of adipsin concentrations for each RD compared to controls

**Figure 2:**
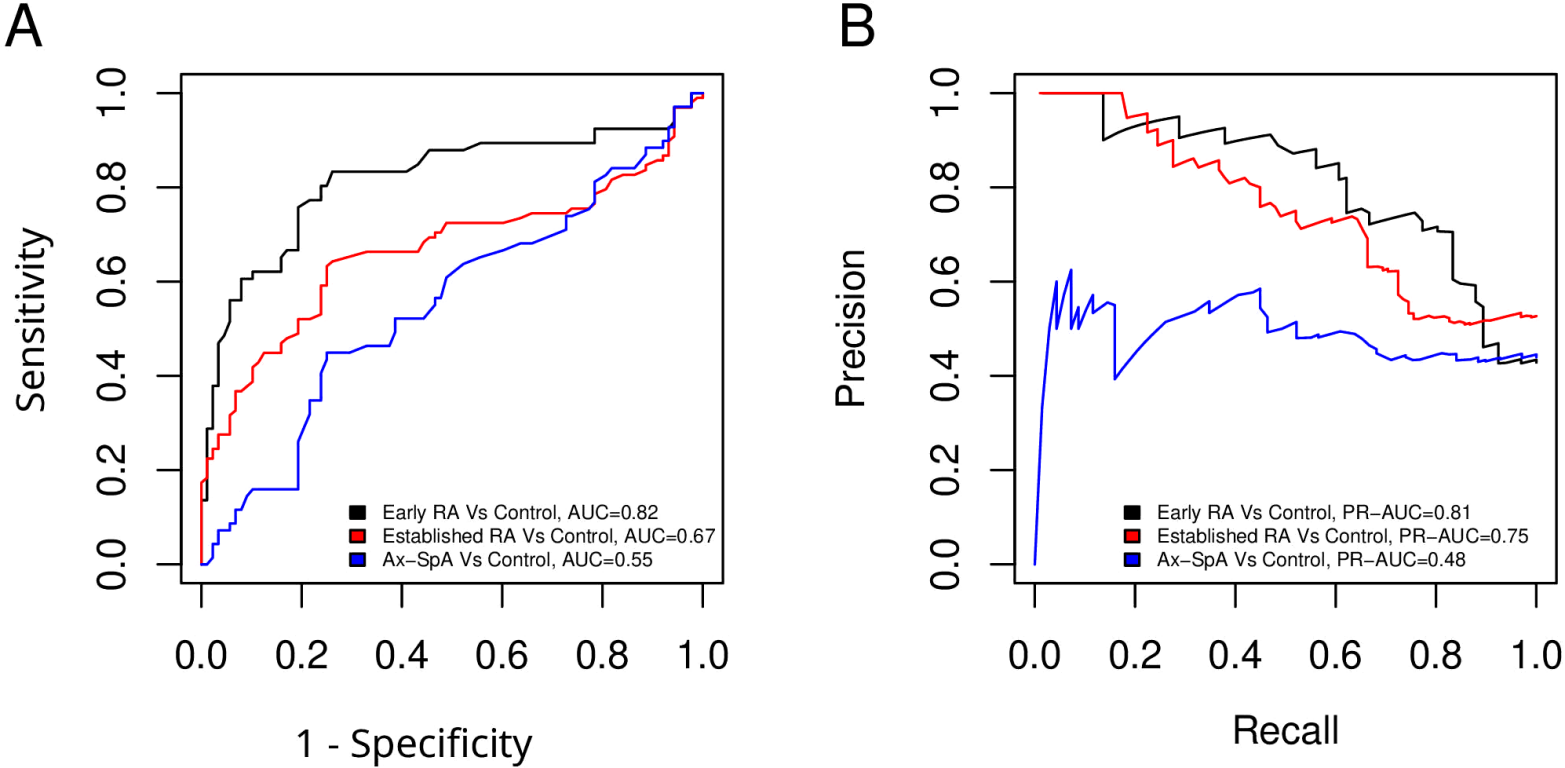
Predictive values of adipsin concentrations for each RD compared to controls. **(A)** ROC curves showing the discriminatory capacity of serum adipsin levels between healthy controls and patients with early-RA, established-RA, and axSpA. AUC values are indicated for each comparison. Optimal cut-offs were defined using the Youden Index. **(B)** Precision-Recall (PR) curves assessing the performance of classification models in distinguishing disease groups from healthy controls. These curves illustrate the balance between precision and recall across different classification thresholds.

A discriminative analysis was performed to evaluate the ability of adipsin levels to distinguish between disease groups, using sensitivity and specificity calculations along with the construction of ROC and PR curves. The results are illustrated in **Figure 2**. The models show high discriminative ability for early-RA (AUC = 0.82; PR-AUC = 0.81) and moderate performance for established-RA (AUC = 0.67; PR-AUC = 0.75). In contrast, performance for axSpA is limited (AUC = 0.55; PR-AUC = 0.48), suggesting reduced discriminatory power for this condition.

## Discussion

This study aimed to explore the role of serum adipsin levels in patients with RDs, specifically RA at different disease stages and axSpA. Our findings reveal notable differences in adipsin concentrations across these groups, thus highlighting its potential relevance as a biomarker in the context of RD.

We observed that early-RA and established-RA patients had significantly higher adipsin levels than patients with axSpA and HCs. These results are in line with studies showing that adipsin levels are associated with disease activity in RA, being particularly higher in early-RA (8, 24). For example, elevated adipsin and leptin levels have been correlated with more marked clinical activity in RA patients, especially in those with coexisting conditions such as obesity and periodontal infections (24). These findings suggest that adipsin may be involved in the inflammatory processes that are characteristic of the early stages of RA, potentially reflecting a heightened immune response or metabolic alteration associated with the disease.

Our regression analyses identified distinct clinical variables associated with adipsin levels in different patient groups. In HCs, we observed a modest yet statistically significant sex-related difference, with males exhibiting higher adipsin concentrations than females. This aligns partially with prior reports suggesting that adipokine profiles may be influenced by sex hormones and fat distribution patterns (25, 26). However, this sex effect appears disease-context dependent, as the trend reversed in early-RA patients, where female sex was associated with significantly higher adipsin levels. This may reflect disease-driven modulation of adipose tissue function or immune-metabolic interactions that differ between sexes in the inflammatory setting.

The correlation between adipsin levels and disease duration in established-RA and axSpA patients suggests that adipsin might play a role in the progression of these diseases, consistent with previous studies indicating that chronic inflammation can modify adipokine profiles, leading to altered serum levels (4, 5, 7, 24). The positive correlation between adipsin levels and age in established-RA patients further indicates that both age and disease progression may influence adipokine dynamics, echoing findings from Valverde-Franco et al., who highlighted the complex interplay between adipokines and progression of RDs (18). Stratification by age tertiles confirmed this pattern, with significantly lower adipsin levels observed in the youngest group (<50 years) compared to older patients. Interestingly, the plateau seen between middle-aged and older patients suggests that adipsin expression may increase to a certain threshold beyond which levels stabilize, possibly reflecting saturation in complement-related activity or metabolic adaptation. The lack of an association between adipsin levels in axSpA and in HCs and, in contrast, the differences observed for early and established-RA underscore the role of the alternative pathway of the complement system in the pathogenesis of RA (27). Importantly, our discriminative analysis revealed that adipsin levels possess diagnostic potential, particularly in distinguishing early RA patients from healthy controls. An AUC of 0.82 with a defined cutoff of 1.325 µg/mL indicates strong sensitivity and specificity, suggesting that adipsin may serve as a valuable adjunct biomarker in the early identification of RA, where timely diagnosis and intervention are critical.

Interestingly, adipsin levels were associated with the use of prednisone in early-RA patients, indicating that treatment with glucocorticoids might enhance adipokine secretion. This observation aligns with the understanding that glucocorticoids can impact metabolic pathways and adipokine production (28, 29). For example, prednisolone has been shown to alter adipokine secretion in 3T3-L1 adipocytes, including the upregulation of leptin and other metabolic regulators, while suppressing inflammatory mediators through pathways such as Wnt and Akt signalling (30). Moreover, a study demonstrated that short-term prednisone administration increased circulating levels of adiponectin and leptin without altering body composition in healthy individuals (31). Clinically, glucocorticoids have been reported to affect adipokine levels and contribute to metabolic disturbances in conditions such as Cushing’s syndrome, RA, and obstructive airway diseases (32–34). Collectively, these findings support the notion that glucocorticoids may play a pivotal regulatory role in adipokine expression, including that of adipsin. Future research should focus on elucidating the mechanisms underlying this relationship and evaluating whether adipsin can serve as a predictive biomarker for treatment response.

Together, these findings suggest that adipsin levels are influenced by multiple demographic and clinical variables, which should be carefully considered when interpreting its potential role in RA pathogenesis.

Although serum adipsin is markedly elevated in both early and established RA -correlating with disease duration and patient age- this observation alone does not establish causality and may simply reflect chronic activation of the alternative pathway of the complement system. In the context of RA, where systemic inflammation is a hallmark, adipsin could contribute to tissue damage by enhancing complement-mediated responses. This activity could perpetuate local inflammation in the joints, amplifying synovial inflammation and promoting osteoclastogenesis. Furthermore, adipsin has been shown to affect various immune cells, including macrophages and dendritic cells, potentially exacerbating the inflammatory milieu (9).

On the other hand, adipsin may also be a downstream consequence of inflammation rather than a primary driver. In RA, systemic inflammation is characterized by the production of pro-inflammatory cytokines such as TNF-α, IL-6, and IL-1β, which are known to influence adipokine secretion (4). It is possible that elevated adipsin levels are a secondary response to these inflammatory signals, serving as a compensatory mechanism to regulate immune function or metabolic processes in the setting of chronic inflammation. This hypothesis is supported by studies in other inflammatory diseases, where adipokines like adiponectin and leptin are upregulated in response to inflammatory stimuli (4, 5).

To further clarify the role of adipsin in RA, future studies should focus on delineating whether elevated adipsin is a direct mediator of inflammation or a consequence of the inflammatory process. Stratified analyses accounting for corticosteroid use, disease duration, and disease activity could help identify whether adipsin levels are independently associated with disease severity or if they merely reflect the underlying inflammatory state. Furthermore, functional studies exploring the effects of adipsin on immune cell activation, cytokine production, and complement activation could provide more definitive evidence for its role in driving inflammation in RA.

However, several limitations should be acknowledged. The cross-sectional nature of this study precludes causal inference, and longitudinal studies are needed to assess the dynamic regulation of adipsin in response to treatment or disease progression. Additionally, although the sample size was adequate for detecting associations, further validation in larger and more diverse populations will strengthen generalizability. Another limitation of our study is the lack of BMI data for the healthy control group, which restricts our ability to fully assess the influence of adiposity on circulating adipsin levels. This is particularly relevant given the known role of adipose tissue in modulating adipokine secretion. Nevertheless, within the patient cohorts (early RA, established RA, and axSpA), we did not observe significant associations between BMI and adipsin concentrations. This suggests that, at least in our population, BMI may not be a major driver of the differences observed. However, we recognize the importance of including BMI-matched controls in future studies to strengthen the interpretation of adipokine-related findings in rheumatic diseases.

In conclusion, while adipsin appears to play an important role in the alternative pathway of the complement system and may influence immune function in RA, its exact role in disease pathogenesis remains to be fully elucidated. Additional research, particularly longitudinal studies and functional assays, is necessary to determine whether adipsin serves as an active driver of inflammation or a downstream consequence of chronic immune activation in RA.

## Data Availability

The original contributions presented in the study are included in the article/supplementary material. Further inquiries can be directed to the corresponding author.
